# Challenging Additivity: Comparing Predicted and Observed AhR Activity of Polycyclic Aromatic Compound (PAC) Mixtures Containing Active and Inactive Constituents

**DOI:** 10.1021/acs.est.5c11914

**Published:** 2026-01-21

**Authors:** Kristin M. Eccles, Kimberly Gaston, Emily M. Green, Suramya Waidyanatha, Billie Stiffler, Shawn F. Harris, Cynthia V. Rider, Elizabeth Medlock Kakaley

**Affiliations:** † Environmental Health Science and Research Bureau, Healthy Environments and Consumer Safety Branch, Health Canada, Ottawa, Ontario K1A 0K9, Canada; ‡ Oak Ridge Institute for Science Education, Oak Ridge, Tennessee 37830, United States; § Division of Translational Toxicology, National Institute of Environmental Health Sciences, Durham, North Carolina 27709, United States; ∥ 42786 Battelle, Columbus, Ohio 43201, United States; ⊥ DLH, LLC, Bethesda, Maryland 20814, United States; # Center for Public Health and Environmental Assessment, Public Health and Integrated Toxicology Division, U.S Environmental Protection Agency, Durham, North Carolina 27709, United States

**Keywords:** chemical mixtures, potency, benchmark concentration, additivity, bioassay, modeling

## Abstract

Class-based cumulative risk assessment approaches have been applied to high-priority environmental contaminants such as polycyclic aromatic compounds (PACs), yet uncertainties remain in their application. In this study, we evaluated the influence of inactive chemicals on mixture modeling outcomes and explored strategies for predicting the aryl hydrocarbon receptor (AhR)-mediated toxicity of PAC mixtures. Using an in vitro AhR reporter gene assay, we tested seven defined mixtures composed of six active and seven inactive PACs. Observed concentration–response curves were compared to predictions from three established mixture models, concentration addition (CA), independent action (IA), and generalized concentration addition (GCA), using both effective concentration eliciting 10% response (EC10) and benchmark concentration (BMC_10_) approaches. Including inactive chemicals without scaling led to consistent overestimation of potency, especially in models assuming equal efficacy. Predictive accuracy improved across all models when mixtures were limited to active chemicals and contributions were scaled to 100%, excluding inactives. Among approaches, GCA consistently produced the best agreement with measured responses, particularly when paired with BMC modeling. BMC10 values better accommodate partial agonists. Our findings support a pragmatic, mechanism-based framework for modeling environmental mixtures, one that prioritizes active components, scales their contributions, and adopts BMC-based methods to estimate potency.

## Graphical abstract

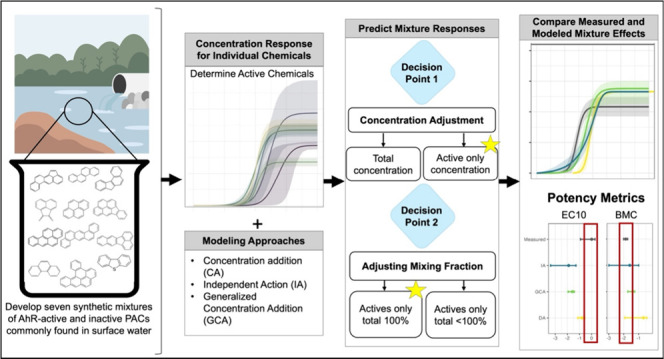

## Introduction

Environmental contaminant exposures do not occur in isolation but as complex and dynamic mixtures. In particular, polycyclic aromatic compounds (PACs), defined as compounds with a minimum of two fused six-atom aromatic rings,^1–3^ are ubiquitous in the environment and are frequently detected as complex mixtures in environmental samples.^4,5^ PACs have been characterized in multiple exposure scenarios, including occupational settings and inhalation exposures.^6,7^ Some members of the PAC class, such as benzo­[*a*]­pyrene, the most well-studied PAC, elicit a broad spectrum of toxicities, including carcinogenicity, developmental toxicity, reproductive toxicity, and immunotoxicity.^8^ Although thousands of structural configurations are possible, environmental monitoring efforts focus primarily on the 16 priority PACs.^9,10^ Furthermore, cumulative risk evaluations of PACs have typically focused on carcinogenicity and included only a limited subset of individual PACs with available in vivo cancer data. Specifically, the relative potency factor approach, a component-based cumulative risk assessment method that is based on an assumption of dose additivity, has been used by the US EPA to evaluate PAC-containing mixtures. Extending cumulative risk approaches to a broader range of PACs and toxicities requires careful testing of hypotheses about joint action with various end points of interest.

Although PACs act through multiple mechanisms and activating the aryl hydrocarbon receptor (AhR) plays a key role in their observed toxicities.^11^ AhR activation has been linked to adverse physiological effects, including tumor growth and survival,^4^ reduced bone growth,^12^ cardiotoxicity,^13^ and subsequent early life stage mortality.^14^ Further, adverse outcome pathways (AOPs) have been developed to causally link AhR perturbation as a molecular initiating event (MIE) to these and other apical end points, including sustained AhR activation leading to rodent liver tumor promotion,^15^ AhR-mediated early life stage mortality via SOX9 repression associated with craniofacial and cardiac malformations,^16^ and a quantitative AOP network connecting AhR activation to lung tissue damage.^17^

High-throughput approaches and/or in vitro screening methods have been used to screen individual PACs and candidate PAC mixtures for toxicity.^1,18–20^ Specifically, in vitro bioassays have been used to screen individual PACs for AhR transcriptional activation^21^ and mixtures in environmental samples, such as surface water, for AhR bioactivity.^22–24^

For PAC mixtures, in vitro AhR transcriptional activation assays may provide information on how the chemicals interact with the AhR individually and in combination, providing valuable insights into the joint action of chemicals. Predictive models of mixture toxicity, such as concentration addition (CA),^25,26^ independent action (IA),^27^ and generalized concentration addition (GCA),^28^ can be used to estimate responses to environmental mixtures based on individual compound concentration–response data. Furthermore, comparing predicted to observed mixture responses enables the detection of less-than-additive or greater-than-additive interactions among constituents.^29^ However, the application of in vitro bioassays to assess complex environmental mixtures containing inactive compounds has, in some cases, resulted in responses not predicted by traditional additive mixture models.^30–32^

Although many studies have compared predicted and measured effects of defined mixtures, few have explicitly examined how inactive chemicals contribute to these responses or influence mixture modeling, particularly in a pre-emptive, mechanistic context. Thus, we aimed to accurately predict the PAC mixture effects on AhR transcriptional activation using models of additivity, which assume no interactions among constituents, and to determine how the inclusion of inactive chemicals in a mixture affects predictions of efficacy and potency. We quantified the effect of inactive chemicals in the mixture by answering the following questions: (1) What, if any, are the impacts of predicting chemical mixtures when inactive compounds are included in the mixture? and (2) What is the best way to account for inactive chemicals in predictive modeling? Throughout this paper, we examine different mixture formulations to assess when and to what extent the modeling decisions of including inactive chemicals influence predicted mixture responses.

## Materials and Methods

### Chemical Selection

We selected 13 different PACs for inclusion in this study (Table S1). Most PACs were selected based on their environmental presence and range of carcinogenic potencies. Two additional PACs were selected to add structural diversity, representing groups not typically included in cumulative risk assessments; dibenzothiophene a sulfur-containing heterocyclic PAC, and acenaphthenequinone an oxygenated PAC.

### Mixture Formulation

We developed seven different mixtures to investigate our research questions (summarized in Figure 1 and Table S2). Only 6 of the 13 PACs in the mixture were “active” in the AhR assay (Figure 1A), defined as having a concentration–response slope statistically different from zero (*p* < 0.05) (Table S3). Mixtures were designed with and without the seven inactive PACs. The first equimolar mixture (EM) contained each of the 13 chemicals, which were intended to be present at equal molar concentrations (Figure 1B,E). However, due to solubility limitations, the final concentrations of some compounds, notably benzo­[*c*]­fluorene and chrysene, were adjusted to their maximum solubility in DMSO, resulting in slight deviations from true equimolarity. Two additional 13-chemical mixtures, labeled experimental mixtures 1 and 2 (EXP1 and EXP2) contained all of the individual PACs at ratios that were based on ratios derived from equipotent levels in complementary in vivo studies that will be published separately. Due to differences in the experimental models (mice toxicity studies versus AhR in vitro assay), EXP1 and EXP2 were not expected to be equipotent in the current study. In EXP1 benzo­[*a*]­pyrene, benzo­[*b*]­fluoranthene, and indeno­[*1,2,3-cd*]­pyrene dominated the mixture (Figure 1C,F), while benz­[*j*]­aceanthrylene, benzo­[*a*]­pyrene, benzo­[*b*]­fluoranthene, and indeno­[1,2,3-*cd*]­pyrene contributed the largest share to EXP2 (Figure 1D,G). The final mixture (EXP3) was designed to be equipotent in the AhR assay based on preliminary analysis of individual chemical data (Figure S1 and Table S3). The ratio in EXP3 differed significantly from EXP1 and EXP2, with benzo­[*a*]­pyrene making up over 70% of the mixture mass (Figure 1K).

**Figure 1:**
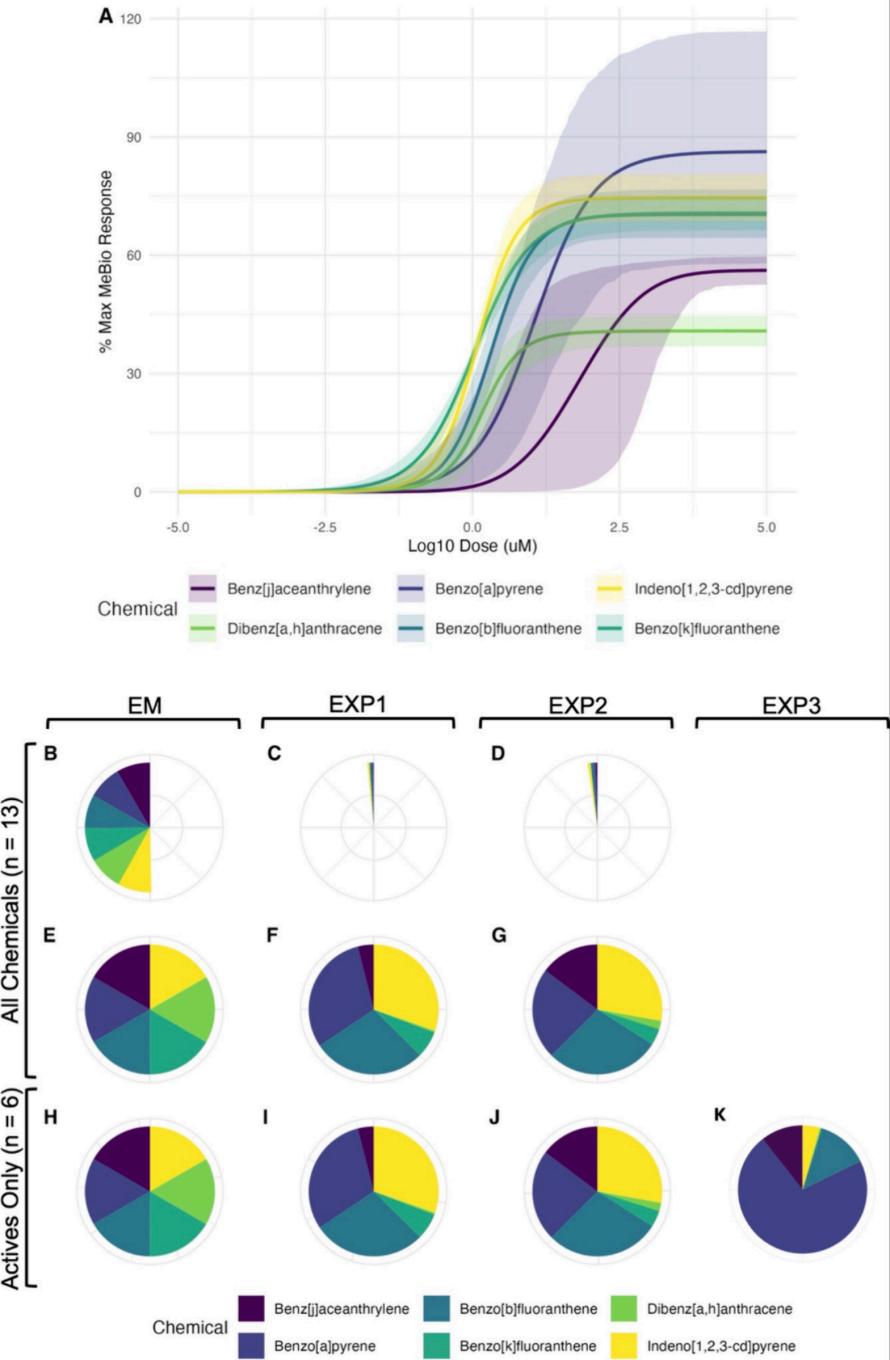
Concentration–response curves in the INDIGO Biosciences Human AhR Reporter Assay System for individual chemicals (Panel A) that were defined as active (i.e., concentration–response slope statistically different from zero) displayed as the percentage of the maximum response relative to the positive control (MeBio, a known AhR agonist) as a function of Log10 concentration (μM). Shaded areas represent the 95% confidence intervals of the curve fits. A summary of the proportion of each active chemical within the mixtures is also provided. Panels B–D show mixtures containing all 13 chemicals, though only the active components are visualized. Panels E–G present the same mixtures as B–D, but with proportions reweighted so that the active components collectively represent 100% of the mixture. Panels H–K display mixtures composed exclusively of the six active chemicals.

### In Vitro AhR Activation Assay

The INDIGO Biosciences Human AhR Reporter Assay System (INDIGO Biosciences, Inc., State College, PA, USA; 1 × 96-well format assay) and manufacturer’s protocol were used to detect in vitro AhR transcriptional activation response of 13 individual PACs and 7 PAC mixtures. Cells were plated on a white, opaque-bottom 96-well plate. After incubation for 4–6 h at 37 °C and 5% CO_2_, cells were either treated with the MeBio standard (a known AhR agonist and positive luminescence control; 0, 0.064, 0.32, 1.6, 8.0, 40, 200, and 1000 nM), test compounds, or defined mixtures dissolved in DMSO, DMSO vehicle control (final DMSO concentration ≤0.4% to minimize cytotoxicity), or 8 μM staurosporine (INDIGO Biosciences, the positive control for cytotoxicity), diluted using Cell Screening Media (INDIGO Biosciences) (Table 1). Additionally, a triplicate of wells with media only (no cells) and wells with cells only (no vehicle) were included on each plate for cytotoxicity determination (see below). Each control and test treatment was run in triplicate, and each test compound/mixture was tested across 2–3 different plates.

**Table 1:** Summary of the Percentage Contribution of Active Compounds Used to Generate the Predicted Mixture Responses

	percentage contribution						
	method 1: contribution unadjusted			method 2: contribution adjusted			
chemical	EM	EXP1	EXP2	EM	EXP1	EXP2	EXP3
benz[*j*]aceanthrylene	8.3	0.1	0.5	16	4	14	11
dibenz[*a,h*]anthreacene	9.1	0.007	0.1	18	0	2	0.2
benzo[*a*]pyrene	8.3	0.6	0.7	16	30	22	72
benzo[*b*]fluoranthene	8.3	0.5	0.9	16	27	28	13
indeno[1,2,3-*cd*]pyrene	9	0.6	1	18	32	30	4
benzo[*k*]fluoranthene	8.3	0.1	0.1	16	6	4	0.3
sum	51.2	1.9	3.3	100	100	100	100

After 22–24 h of incubation (37 °C, 5% CO2), the Live Cell Multiplex Cell Assay (LCMA; INDIGO Biosciences, 1 × 96-well format assays: LCMA-01) cytotoxicity was quantified using the manufacturer’s protocol (eq 1).

$$\%\;\mathrm{Live}\;\mathrm{Cells}=\frac{\mathrm{Mean}\;{\mathrm{RFU}}^{-\mathrm{BKG}}\;\mathrm{of}\;\mathrm{treated}\;\mathrm{cells}}{\mathrm{Mean}\;{\mathrm{RFU}}^{-\mathrm{BKG}}\;\mathrm{of}\;\mathrm{untreated}\;\mathrm{cells}}\times 100$$ 1

Any treatment concentration resulting in ≥15% reduction in live cells compared to untreated cells was considered cytotoxic and excluded from further analysis.

LCMA fluorescence (relative fluorescence units: RFU) and luminescence (AhR activation, relative light units; RLU) were quantified in succession using a ClarioSTAR luminometer (BMG Labtech CLARIOstar software, V4.01 R2).

Using GraphPad Prism v8.0 (GraphPad Software, La Jolla, California), RLU values were transformed as previously described.^33,34^ Briefly, RLU values for all individual PACs, PAC mixtures, and MeBio were normalized to the DMSO vehicle control (fold induction) mean, and exposure concentrations were log10-transformed. Each chemical or mixture treatment was normalized to the maximal response of the MeBio standard curve, converting the fold change into a percent maximal response of the MeBio reference chemical.

We evaluated the quality of our assay data using a *Z*-factor, which is a dimensionless parameter that indicates a degree of confidence in reporting positive hits/responses, by comparing the signal-to-noise and signal-to-background values of the in vitro screening assay.^35^ *Z*-factor values range from 0 (unacceptable assay) to 1 (ideal assay), where an assay with a *Z*-factor > 0.5 is classified as excellent and <0.5 is classified as marginal. The *Z*-factor for our AhR assay was 0.840, using the MeBio (manufacturer’s suggested positive control) concentration–response data. These results indicated both a high dynamic range and low data variation for the data used in this study.

### Concentration Response Modeling for Individual Chemicals

Individual PAC concentration–response curves were fit using a 4-parameter (eq 2) logistic hill equation using the drm (drc package^36^) in R v4.3.1; all subsequent curve fitting and model-based predictions were conducted in R.

$$f(x)=c+\frac{d-c}{1+\exp(b(\log(x)-\log(e)))}$$ 2

Here, the response *f*(*x*) is predicted using *x*, the chemical concentration, where *c* is the minimum response at the bottom of the curve, *d* is the maximal response at the top of the curve, *b* is the slope, and *e* is the effective concentration at halfway between the baseline and maximal responses (EC_50_). In all models, the bottoms of the curves (*c*) were constrained to zero.

Benchmark concentration modeling was conducted to estimate the concentration at which mixtures elicited a 10% increase in AhR activation above zero (BMC_10_). Predicted and measured concentration–response data were modeled using a Hill function implemented through the concRespCore function in the tcplfit2 R package.^37^ A 10% benchmark response (BMR) was specified as 10 units from the baseline response of zero (tcplfit2; bmed = 0, onesd = 10). BMR values were applied as absolute increases from baseline (bmr_scale = 1). All models assumed unidirectional responses and used a normal error distribution (errfun = “dnorm”) to match the error distribution used in the drc package. The EC_10_ estimate from the tcplfit2 package matched the EC_10_ estimate obtained from the drc package, where EC_10_ is the effective concentration at 10% of the maximal response.

### Mixture Modeling

We explored two methods for calculating the mixing fractions to incorporate into the predicted effect of the mixture. Prior to this, chemicals were classified as inactive if the 95% confidence interval for the slope parameter included zero, and were excluded from mixture modeling. In one set of models, we included all chemicals (active and inactive) in the mixing ratios (i.e., active chemicals <100% of the total mixture) (Figure 1B–D and Method 1 in Table 1). In the second set of models, we recalculated the mixing proportions, excluding the inactive chemicals so that the mixing fractions of the active chemicals added up to 100% (Figure 1E–G and Method 2 in Table 1). To meet the model assumptions of CA and IA (summarized in Table S4), which state that all concentration–response curves have the same minimal and maximal effects, we refit the active chemicals (eq 2), fixing the top of the curve to the maximum response of the active chemicals, which was 73.02%. In order to meet the model assumptions of GCA, we refit the active chemicals again, fixing the slope to 1. Each mixture modeling method is further detailed below, and the fit parameters are listed in Table S5.

The confidence intervals for each mixture concentration–response parameter (slope, EC_50_, and top of the curve) were determined by generating 1000 parametric bootstrap values sampled from the standard error values of each parameter. The values were generated to fit a truncated random normal distribution for the EC_50_ and the top of the curve, thereby avoiding negative values. The bootstrapped values were then fit to a random normal distribution for the slope. This method for generating the confidence intervals assumes that the parameters are normally distributed and independent of one another.

We tested three mixture prediction methods: CA, IA, and GCA. For each individual chemical, the parameters for the Hill curve (slope, EC_50_, and top-of-curve) were sampled 1000 times using the mean and standard error values estimated from their model fits. For the EC_50_ and the top-of-curve parameters, the values were sampled based on a truncated random normal distribution, thereby avoiding negative values. For the slope parameter, the values were sampled from a random normal distribution. These 1000 parameter values were then used to create the 1000 prediction curves by applying the mixture modeling methods described below. Finally, model fits of these 1000 curves yielded parameter estimates for the mixtures, and the confidence intervals for these parameters were reported as the 2.5% and 97.5% quantiles of the 1000 estimates.

CA is shown in eqs 3 ^38^ and 4:^25^

$$\sum_{i=1}^{n}\frac{C_{i(\mathrm{mix})}}{\mathrm{EC}{x^{*}}_{i}}=1$$ 3

Here, *C*_*i*(mix)_ is the concentration of the *i*th individual chemical within the mixture at *x* effect of the whole mixture, and EC*x* ^*^_i_ is the concentration of the *i*th individual chemical alone that would elicit the same effect level (*x*) as the mixture. eq 3 can be rearranged to include the proportion of each chemical in the mixture (eq 4).^25^

$$\mathrm{EC}x_{\mathrm{mix}}={\left(\sum_{i=1}^{n}\frac{p_{i}}{\mathrm{EC}x_{i}}\right)}^{-1}$$ 4

Here, EC*x*_mix_ is the effective concentration of the mixture, whereby the sum of the ratios between *p*_*i*_ is the proportion of chemical *i* present in the chemical mixture, and EC*x*_*i*__,_ which is the individual chemical concentration that elicits the same response as the mixture. We use an EC_50_ obtained by fitting eq 2 to the mixtures presented in this paper.

IA is given by eq 5:^27^

$$R_{\mathrm{mix}}=1-\prod_{i=1}^{n}(1-R_{i})$$ 5

Here, *R*_mix_ is the mixture response, where *n* is the number of observed chemicals in the mixture, and *R*_*i*_ is generated using the individual concentration–response curves as given in eq 2. Since this method is probability-based, the output values are between 0 and 1. To rescale this output response to the magnitude of this data set, a weighted average of the maximum efficacy (top of the curve) for all active chemicals in the mixture was weighted by the chemical contribution to the mixture (percent contribution: Table 1, efficacy (Top): Table 2). We use a similar method to rescale CA.

**Table 2:** Concentration-Response Parameters for Individual Chemicals and Mixtures, Including % Efficacy (Top) and Effective Concentration at the 50% Maximal Effect (EC50), Along with Their 95% Confidence Intervals (95% CI)^a^

	slope			top (%)			EC50		
chemical	value	*p*-value	95% CI	value	*p*-value	95% CI	value	*p*-value	95% CI
**acenaphthenequinone**	**0.98**	**0.64**	**3.18–5.14**	**6.68**	**0.64**	**–56.62**	**5.58**	**0.83**	**–45.62–56.77**
benz[*j*]aceanthrylene	1.31	<0.001	0.87–1.74	56.28	<0.001	52.23–60.32	0.85	<0.001	0.59–1.09
benzo[*a*]pyrene	0.92	<0.001	0.53–1.29	86	<0.001	54.24–117.76	2.76	0.05	–0.01–5.52
benzo[*b*]fluoranthene	1.2	<0.001	0.85–1.54	70.29	<0.001	63.47–77.09	0.47	<0.001	0.32–0.62
**benzo[** *c* **]fluorene**	**1.37**	**0.52**	**–2.85–5.58**	**14.84**	**0.66**	**–51.84–81.52**	**0.78**	**0.78**	**–4.71–6.28**
benzo[*k*]fluoranthene	0.92	<0.001	0.7–1.13	70.69	<0.001	66.39–74.98	0.04	<0.001	0.02–0.05
**chrysene**	**1.17**	**0.22**	**–0.71–3.05**	**32.28**	**0.55**	–75.69–140.25	**1.06**	**0.72**	**–4.77–6.88**
dibenz[*a,h*]anthracene	1.4	<0.001	0.71–2.09	40.67	<0.001	36.61–44.73	0.01	<0.001	0.007–0.017
**dibenzo[** *a,l* **]pyrene**	**0.49**	**0.88**	–5.77–6.76	**1.96**	**0.8**	**–13.2–17.12**	**0.04**	**0.95**	**–1.45–1.54**
**dibenzothiophene**	**0.05**	**0.98**	**–3.54–3.64**	**1.08**	**NA**	**NA**	**0.58**	**NA**	**NA**
indeno[1,2,3-*cd*]pyrene	1.28	<0.001	0.89–1.67	74.6	<0.001	67.96–81.23	0.15	<0.001	0.11–0.18
**phenanthrene**	**0**	**NA**	**NA**	**0.78**	**0.74**	**–3.91–5.48**	**12.48**	**NA**	**NA**
**pyrene**	**2.23**	**NA**	**NA**	**6.08**	**NA**	**NA**	**644.24**	**NA**	**NA**
EM actives only	–1.44	<0.001	0.86–2	54.14	<0.001	48.77–59.5	0.04	<0.001	0.02–0.05
EM all chemicals	–0.81	<0.001	0.65–0.96	89.72	<0.001	85.79–93.65	0.01	<0.001	0.01–0.01
EXP1 actives only	–1.34	<0.001	0.84–1.84	69.77	<0.001	61.46–78.06	0.1	<0.001	0.06–0.13
EXP1 all chemicals	–1.74	<0.001	0.66–2.81	39.64	<0.001	34.3–44.97	3.59	<0.001	2.08–5.10
EXP2 all chemicals	–1.52	<0.001	0.8–2.23	49.59	<0.001	43.88–55.3	3.56	<0.001	2.23–4.88
EXP2 actives only	–1.68	<0.001	0.75–2.61	33.68	<0.001	29.21–38.15	0.04	<0.001	0.01–0.05
EXP3 actives only	–1.49	<0.001	1.01–1.96	74.49	<0.001	69.08–79.88	0.59	<0.001	0.43–0.74

a In the equimolar (EM) and experimental mixtures (EXP1-3), Actives Only indicates mixtures containing only active chemicals (*n* = 6), and All Chemicals indicates mixtures containing all chemicals (*n* = 13). NA values indicate cases where parameters could not be calculated due to poor model fit; these are bolded to indicate they were not included in the actives-only mixtures.

However, both CA and IA are limited in their ability to predict the joint effect of chemical mixtures where partial agonists are present. Since individual chemicals display partial agonism in that not all chemicals reach the same maximum effect level (Figure 1), we also included the GCA method for estimating the mixture effect, which relaxes the assumption that all chemicals in the mixture have the same efficacy.^28^ GCA is given in eq 6:

$$\sum_{i=1}^{n}\frac{C_{i(\mathrm{mix})}}{f_{i}^{-1}(x)}=1$$ 6

Here, *C*_*i*(mix)_ is the concentration of the *i*th individual chemical within the mixture at *x* effect of the whole mixture, and *f*_*i*_^–1^(*x*) is the inverse of the function describing the concentration–response relationship for *i*. This method assumes that all concentration–response curves have the same slope; thus, using eq 2, the slope is fixed to 1 for all chemicals.

Summary metrics of the predicted 4-parameter Hill model were generated to compare with the measured 4-parameter Hill model fits, including slope, top of the curve, EC_10_, EC_50_, and BMC_10_. For the estimated mixture responses, these metrics were derived from 1000 bootstrapped curve fits, with the 2.5th, 50th (median), and 97.5th percentiles used to summarize the distribution of parameter estimates. In contrast, the metrics for the measured responses were based on fitted curve parameters with associated 95% confidence intervals, capturing the uncertainty in the observed data. A model was considered predictive if the 95% confidence interval of the measured response overlapped with the central 95% range (2.5th to 97.5th percentile) of the predicted values. This approach allows for a consistent comparison of central tendencies and variability between the measured and model-estimated mixture responses. All plots were generated in R using the ggplot2 package.^39^

## Results

### Individual Chemicals

We were able to generate concentration–response curves for six out of the 13 tested chemicals, including benzo­[*a*]­pyrene, benzo­[*k*]­fluoranthene, dibenz­[*a,h*]­anthracene, benz­[*j*]­aceanthrylene, benzo­[*b*]­fluoranthene, and indeno­[1,2,3-*cd*]­pyrene (Figure 1A and Table 2; not bold). The remaining chemicals (phenanthrene, pyrene, acenaphthenequinone, benzo­[*c*]­fluorene, dibenzo­[*a,l*]­pyrene, dibenzothiophene, and chrysene) were found to be inactive because the 95% confidence interval for the slope parameter included zero and were therefore excluded from subsequent mixture predictions (Table 2; bold).

### Mixtures

We evaluated seven different mixtures in vitro (Figure 2) with mixtures containing all chemicals (including inactive ones) depicted with green lines and those containing only active PACs presented as blue lines. For mixtures EXP1 and EXP2 (Figure 2), there were no statistical differences in potency between the “all chemicals” and “active chemicals only” mixtures. In contrast, the equimolar mixture (EM) showed greater potency when inactive chemicals were included (Figure 2B; top panel). Across all mixtures, efficacy (top of the curve percentage) did not overlap, and no overarching trend was observed (Figure 2B; middle panel). Although active chemicals had greater efficacy in EM and EXP2 mixtures, all chemical combinations had greater efficacy in the EXP1 mixture. The slopes were not significantly different, as indicated by overlapping 95% confidence intervals (Figure 2B; bottom panel). Notably, most mixtures had a slope near 1, suggesting that the GCA assumption is appropriate in these cases.

**Figure 2:**
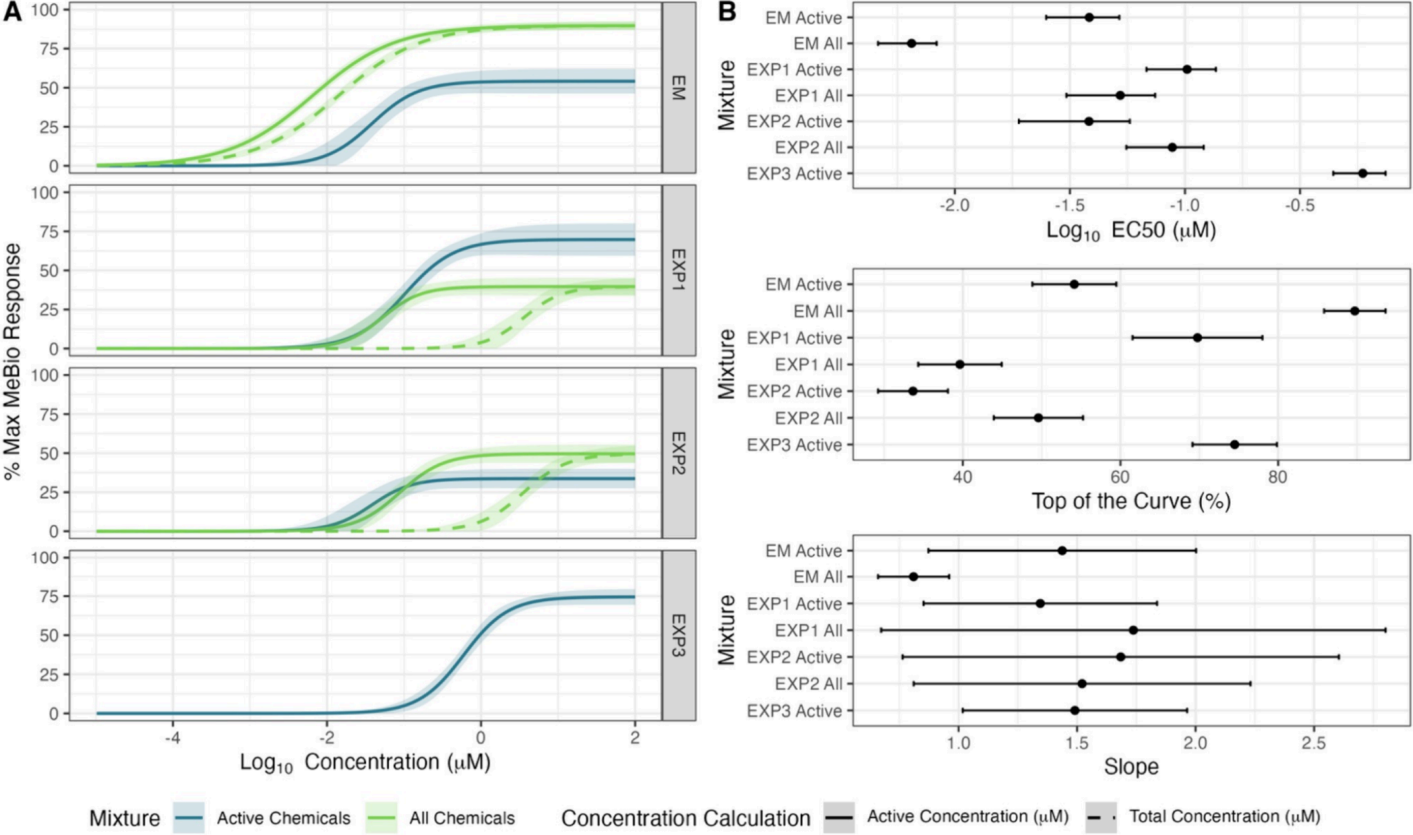
(A) Concentration–response curves for different mixtures (EM, EXP1, EXP2, and EXP3) show the percentage of the maximum MeBio response as a function of log10 concentration (μM). Blue lines are mixtures that only contain the six active chemicals (*n* = 6), while green lines contain all chemicals (*n* = 13). Solid lines represent the concentration–response curves generated with the summed concentration of active chemicals only, while dashed lines represent curves generated with the summed concentration of all 13 chemicals. Shaded regions indicate confidence intervals. (B) Summary plots of key concentration–response parameters, including Log10 EC_50_ (top), maximum response at the top of the curve (middle), and slope (bottom) for each mixture, where all refers to *n* = 13 chemicals and active refers to *n* = 6 chemicals. Data points represent mean values; error bars indicate 95% confidence intervals.

We also assessed how different approaches for calculating the total concentration affect the mixture response. We compared concentration–response curves with the sum of all constituents versus only the active ones for in vitro mixture exposure treatments containing both active and inactive components (Figure 2A; dashed vs solid green lines). Summing the concentrations across all chemicals consistently resulted in a less potent EC_50_; however, this difference was much more pronounced in EXP1 and EXP2 mixtures. The seventh mixture (EXP3 Figure 2A) consists solely of active chemicals and has no “all chemical” counterpart.

To estimate the mixture response, we explored two methods for calculating the percent contribution of each chemical to the predicted mixture effect in mixtures containing all 13 chemicals. Each chemical and its corresponding concentration–response parameters are shown in Table 1. When the contribution was not adjusted, active components comprised 51.1% of the EM mixture, 1.9% of the EXP1 mixture, and 3.3% of the EXP2 mixture (Figure 1A–C and Table 1). In the second method, we adjusted the percent contribution so that the active chemicals accounted for 100% of the mixture (Figures 1D–F, 3B, and Table 1). Dibenz­[*a,h*]­anthracene, the most potent chemical (EC_50_ = 0.01 μM), contributed a small proportion to all mixtures, with the highest proportion observed in EXP2 at just 2%. In contrast, benzo­[*a*]­pyrene, the least potent chemical (EC_50_ = 2.76 μM), had a more substantial contribution across all mixtures.

**Figure 3:**
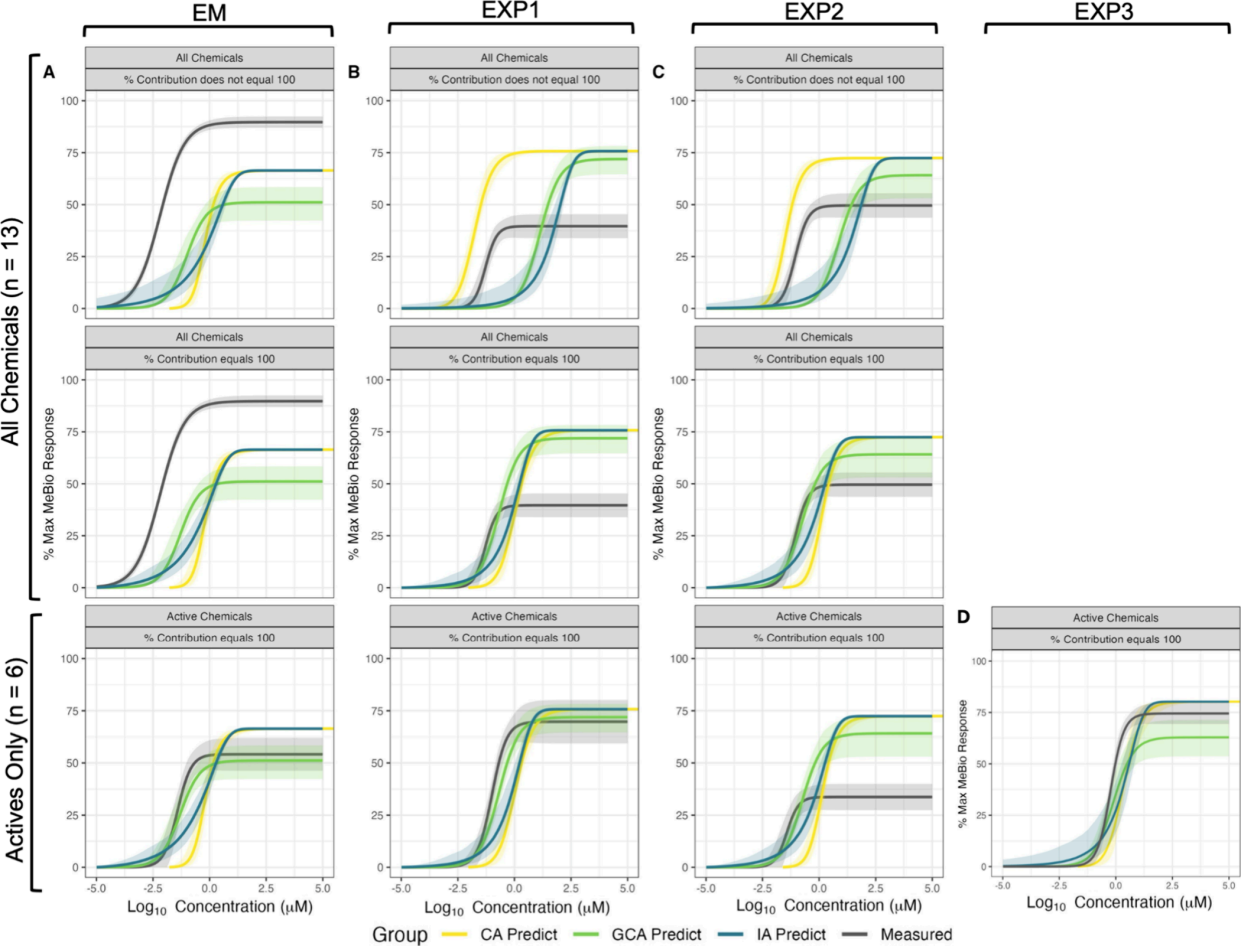
Concentration–response curves comparing measured data (gray) with model predictions using Concentration Addition (CA, yellow), Generalized Concentration Addition (GCA, green), and Independent Action (IA, blue). Each panel represents different mixture conditions based on n chemicals in the mixture and concentration adjustments: (A) Equimolar mixture (EM), (B) Experimental Mixture 1 (EXP1), (C) Experimental Mixture 2 (EXP2), and (D) Experimental Mixture 3 (EXP3). Within each panel, subplots compare responses for active chemicals only and all chemicals, with and without contribution adjustments, where appropriate. The shaded regions represent the 95% confidence intervals around the predicted and measured data.

When we compared the observed mixture responses to the model-predicted responses (Figure 3), overall, the observed and predicted curves were most similar in the mixtures where the total contribution of the individual chemicals was equal to 100%. The exception is the equimolar mixture, where the mixture containing all chemicals (*n* = 13) was more potent than the mixture containing only the active components. In general, mixtures where the percent contributions of the active chemicals did not add up to 100% showed the largest differences between the predicted and measured mixture responses. Across all mixtures, those composed only of active components (*n* = 6, a subset of the 13 total components), where the component contributions totaled 100%, were best predicted by GCA (Figure 3).

The curve fit parameters for all mixtures (both predicted and measured) are summarized in Figure S2. In many cases, the slopes were similar (i.e., close to 1) and not statistically different from one another. The tops of the predicted curves were similar, as weighting the top of the curve proportionally to the amount of chemical in the mixture produced results more like those of the GCA method in all mixtures. In some instances, the top of the observed concentration–response curves were significantly lower than the top of the predicted curves. This pattern was not consistent across mixtures, and no overarching trend was observed when comparing tops or slopes across mixtures or models. The measured mixtures were generally more potent than the predicted EC_50_ of the mixture. However, the efficacies of the measured mixtures were generally lower than what we predicted the efficacy of the mixture would be (50% of maximum efficacy), meaning that the EC_50_s are not directly comparable. Since we are using a hill curve fit for all concentration–response models in this research, curves with a lower top of the curve (less efficacious) tended to be more potent than curves with a higher top of the curve (more efficacious), which explains some of the differences in EC_50_ values between measured and predicted responses.

We compared potency at a 10% effect level using BMC_10_ and EC_10_ approaches to evaluate model performance across the PAC mixtures (EM, EXP1, EXP2, and EXP3) and three predictive models: CA, GCA, and IA. Figure 4 shows predicted and measured values for BMC_10_ (panels A and C) and EC_10_ (panels B and D). Panels A and B include all chemicals in the mixtures, with the top row showing unadjusted contributions and the bottom row showing normalized contributions (scaled to 100%). Panels C and D are limited to active chemicals. Across all scenarios, GCA predictions align most closely with measured values, particularly in panels C and D, where only active chemicals are considered. IA shows intermediate performance, while CA overestimates BMC_10_ and EC_10_, reflecting lower predicted potency. This overestimation by CA is especially pronounced in EXP2 and EXP3 mixtures, where CA predictions diverge significantly from measured values. Normalizing contributions and excluding inactive chemicals improves predictive accuracy, with GCA showing the best overall agreement.

**Figure 4:**
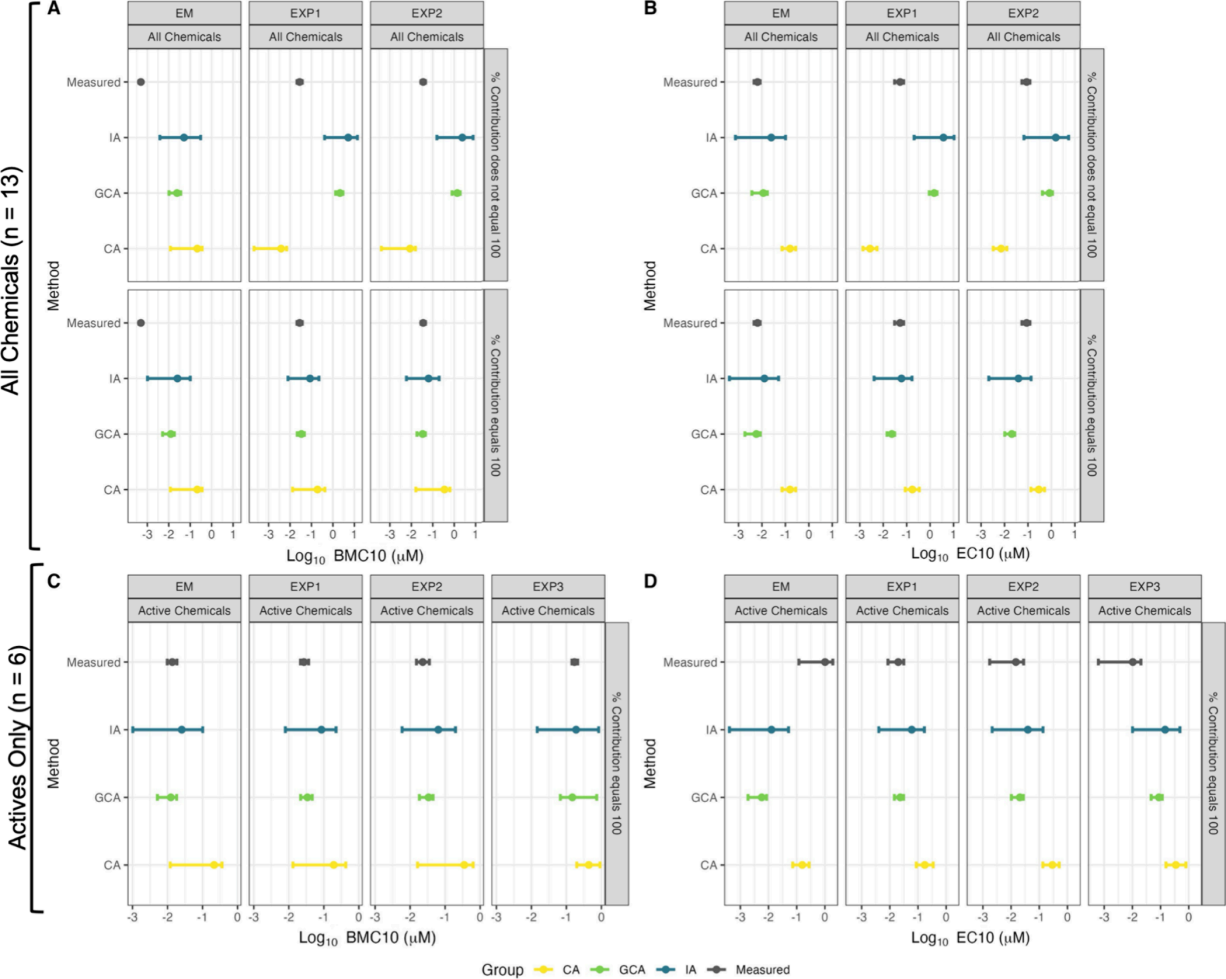
Comparison of benchmark concentrations (BMC_10_), which correspond to the concentration eliciting 10% of the maximal effect above the baseline (A, C), and effective concentrations at 10% maximal response (EC_10_) estimated for the PAC mixtures (EM, EXP1, EXP2, and EXP3) using different predictive models, Concentration Addition (CA), Generalized Concentration Addition (GCA), and Independent Action (IA), versus the measured values. A and B show results for mixtures containing all chemicals under two contribution schemes, where active chemical contributions are scaled to 100%, while the bottom panels show unadjusted contributions. C and D show results for mixtures containing only active chemicals, where contributions are scaled to sum to 100%. Points represent the median EC_10_ or BMC_10_ (log10-transformed), and error bars indicate 95% confidence intervals.

While the BMC_10_ and EC_10_ approaches yield similar trends across models (Figure 4), they differ in interpretability and precision. BMC_10_ represents a fixed increase above the baseline, making it more directly comparable across mixtures, even when maximum effects differ. In contrast, EC_10_ reflects 10% of each mixture’s maximal effect, which can lead to inconsistencies when efficacy varies, particularly when nonlinear models are used. This is illustrated in Figure 4, where EC_10_ predictions (panel D) appear slightly more variable across mixtures than BMC_10_ predictions (panel C), despite tighter confidence intervals. Although EC_10_ estimates tend to have narrower confidence intervals, BMC_10_ provides a more stable reference point for cross-mixture comparisons. Therefore, BMC_10_ may be the more appropriate metric for comparing potency across mixtures with differing top responses.

## Discussion

Efforts to predict the toxicological effects of environmental mixtures have traditionally relied on CA and IA models applied to mixtures composed of well-characterized, active chemicals.^40–42^ Component-based risk assessment approaches, such as the relative potency factor approach commonly applied to chemical classes such as PACs, include some inactive chemicals (e.g., phenanthrene and pyrene^43^), but there is little data available to understand how inactive chemicals affect predictions of additivity. An alternative, albeit a posteriori, approach to address more realistic exposure scenarios is in vivo exposure to whole samples^44,45^ or analyzing in vitro responses to environmental mixtures using extracted samples.^32,46^ A growing challenge in mixture modeling is determining how inactive constituents affect prediction accuracy and whether they should be included in modeling efforts.

Our study found that including inactive chemicals resulted in poorer model fits and lower predictive accuracy. Further, inactive chemicals were included in mixtures, but the percent contributions of active chemicals were not adjusted to 100%, model predictions diverged substantially from measured responses, particularly in terms of potency. This discrepancy emphasizes the importance of contribution adjustment. Mixtures where the contributions of active chemicals were scaled to sum to 100% consistently yielded more accurate predictions, especially when using GCA. The greater accuracy of GCA compared to CA and IA is not unexpected, as GCA models are most appropriate for partial agonists, which was the case with PACs in the AhR assay. Regardless, this suggests that even when inactive chemicals are present, efforts to predict mixture effects should prioritize the active constituents, consistent with recommendations from previous studies.^47–49^

Interestingly, although most experimental mixtures followed predictable patterns, the EM showed a unique result, where including inactive chemicals increased potency relative to the active-only mixture. This divergence could result from interactions between chemicals at equimolar ratios, including potential competitive effects, although in this case, if the inactive chemicals truly do not bind the AhR at the tested concentrations, classical competition would not be expected. Instead, solubility shifts, or low-level biological activity not captured in individual tests, could be contributing. It also highlights that the mixture ratio, not just the mixture composition, can alter biological responses and should be considered in mixture study designs.

Traditional CA and IA models require consistent top and bottom values across individual concentration–response curves. To meet these assumptions, we fixed the top of all fitted curves to the average maximum observed efficacy (73%). However, this approach can misrepresent chemicals that act as partial agonists, such as dibenz­[*a,h*]­anthracene, which had a much lower actual efficacy. Forcing partial agonists to match the response range of full agonists introduces error into the model fits and, ultimately, into the mixture predictions. This highlights a limitation of EC-based approaches, such as EC_50_ or EC_10_, which benchmark from the top of the curve. When curves have different maximum responses and nonlinear models are used to fit the data, comparing EC_*x*_ values across chemicals or mixtures becomes problematic and may lead to inaccurate conclusions. While linear modeling approaches have been proposed as an alternative for low-effect-level comparisons,^50^ we found that the receptor-mediated responses observed in our AhR assay were better suited to nonlinear Hill modeling, which captures key inflection points and sigmoidal response behavior.

To address this, we also evaluated benchmark concentration modeling at a 10% effect (BMC_10_), which benchmarks from the bottom of the curve, an inherently more consistent point across all chemicals and mixtures. While our current implementation utilizes Hill models via the tcplfit2 framework, unlike EC-based methods, BMC approaches do not require equal efficacy. They may therefore be better suited to accommodate the true, non-linear, shape of each chemical’s concentration–response curve. In principle, BMC methods are flexible and can incorporate alternative curve-fitting models (e.g., exponential, polynomial, or linear) and, in certain biological applications, may accommodate curve shapes without a defined top (e.g., toxicogenomics^51,52^ ). As expected, BMC_10_ estimates aligned more closely with measured mixture responses, particularly when using GCA with contribution adjustment. This supports the growing use of BMC methods in regulatory toxicology and risk assessment, especially within NAM-based methods where quantitative potency, rather than maximum effect, is the key concern.^53^

From a risk assessment perspective, potency is more important than efficacy, as evidenced by the use of relative potency factors in risk assessment.^54–57^ Furthermore, regulatory thresholds and hazard benchmarks are typically set based on concentrations that elicit defined low-level responses (e.g., BMC_10_ or EC_10_), rather than on whether a chemical can produce a maximal effect.^58–60^ Although efficacy is important for ensuring model assumptions are met under traditional frameworks, it plays a lesser role in decision-making. As BMC-based approaches become more common, the need to normalize the efficacy may be eliminated, allowing for more accurate and flexible modeling, especially when partial agonists are present.Additionally, the choice of benchmark response level (e.g., BMC_10_ vs BMC_20_ or BMC_50_) may influence mixture predictions, because BMR determines the point on the concentration–response curve used to derive potency. Lower BMRs are closer to background noise and may introduce more uncertainty, while higher BMRs fall in a more stable but steeper region of the curve, possibly improving precision. This can affect how individual chemicals contribute to the overall mixture prediction, especially under additive models that scale by BMC. The domain of applicability of our modeling approach is best suited to defined mixtures composed of structurally related compounds, such as PACs, for which mechanisms of action are at least partially understood and individual concentration–response data are available.

Beyond modeling considerations, this work has practical relevance for environmental monitoring. In vitro bioassays, such as the AhR transcriptional activation assay used here, are part of tiered water quality monitoring programs in the U.S.^61^ and the U.K.^62^ that leverage in vitro bioassays as screening tools that trigger follow-up chemical analyses. In this context, understanding how inactive compounds influence total bioactivity is essential. Inactive chemicals may interfere through antagonism, sorption, or other nonspecific interactions that influence assay sensitivity and reliability.^63–65^ Thus, although focusing on active drivers is pragmatic, inactive constituents still warrant consideration to avoid over- or underestimating hazard potential.

A limitation of our study is the assumption that nominal concentrations reflect bioavailable exposure. Some PACs are highly hydrophobic and prone to binding to plastic surfaces, which may reduce the effective concentration reaching cells in vitro and complicate the interpretation of response data.^66^ For example, dibenzo­[*a,l*]­pyrene, a very hydrophobic PAC (log *P* = 7.71 ^67^), is known to be a potent genotoxicant and carcinogen in vivo, at least partially through an AhR-mediated mechanism,^68^ but was inactive in the AhR assay despite testing up to 27.1 mM. Future studies could improve accuracy by incorporating internal dose measurements or applying chemical disposition models to better estimate the tissue exposure concentration.

Our findings are most relevant to in vitro bioassay-based screening and regulatory applications involving mixtures of known composition and shared pharmacodynamic behavior, such as AhR activation. However, the approach is less applicable to environmental samples with unknown or poorly characterized constituents. It is also limited when mixtures involve divergent mechanisms of action or significant matrix effects (e.g., solubility, bioavailability). Furthermore, modeling frameworks that assume additivity (CA, IA, GCA) may not be appropriate when synergistic or antagonistic interactions occur. As such, while our framework provides valuable insight into defined mixture responses under controlled conditions, its predictive capacity may diminish outside this well-characterized chemical and mechanistic space.

Our findings are based on defined mixtures with known concentrations and shared mechanisms of action; however, real-world environmental samples often contain poorly characterized or unidentified chemicals at low concentrations, many of which may act through distinct or overlapping pathways. This variability in both composition and mode of action may reduce the predictive accuracy of the models applied here. The over- and under-predictions observed in Figure 3 further highlight that mixture-specific factors, such as chemical ratios, background variability, or emergent interactions, can significantly influence outcomes. These complexities introduce uncertainty when extrapolating to environmentally relevant mixtures. Future studies should evaluate the robustness of these modeling approaches with more complex mixtures, assess how benchmark response levels affect predictions, and extend this framework to mixtures involving diverse biological targets and mechanisms of action.

In conclusion, this study offers new insight into how inactive constituents influence mixture toxicity by isolating their effects on AhR activation and predictive modeling within a defined chemical class. Our findings support a pragmatic approach to environmental mixture modeling, prioritizing the most active chemicals, scaling their contributions, and utilizing BMC-based methods to estimate potency. This strategy improves both the accuracy and interpretability of predictions, particularly in regulatory contexts where potency is a key parameter for risk assessment. By comparing traditional EC-based approaches with benchmark concentration modeling, we demonstrated that BMC methods offer more reliable predictions, especially when paired with GCA, without requiring uniform efficacy across chemicals. Our results also demonstrate that including inactive chemicals without contribution adjustment can lead to inaccurate model outputs, whereas adjusting the contribution to include only active constituents improves predictive alignment with observed responses. Although no single approach can fully capture the complexity of environmental mixtures, especially those with unknown components or multiple modes of action, our work provides a framework for improving the design and interpretation of mixture studies. This is particularly relevant for in vitro assays, which are increasingly used in environmental monitoring programs, where rapid, mechanism-based screening must balance simplicity and scientific rigor.

## Supplementary Material



## Notes

Preliminary concentration–response curves for individual polycyclic aromatic compounds (PACs); detailed results for equimolar and experimental mixtures (EM, EXP1–EXP3) comparing measured responses with concentration addition (CA), independent action (IA), and generalized concentration addition (GCA) model predictions; effective concentration values used to define mixture composition; chemical identities, sources, purity, and solubility constraints; mixture composition tables for all experimental designs; and parameter constraints and fitted concentration–response model parameters for individual PACs (PDF)
