# Supplementary material for: Challenging Additivity: Comparing Predicted and Observed AhR Activity of Polycyclic Aromatic Compound (PAC) Mixtures Containing Active and Inactive Constituents
Source: Environ Sci Technol. 2026 Jan 21;60(4):2976–87. doi: 10.1021/acs.est.5c11914 (PMC12874505; doi:10.1021/acs.est.5c11914)
Supplement: Supplementary file 1 [file es5c11914_si_001.pdf]

Challenging Additivity: Comparing Predicted and Observed AhR Activity of Polycyclic Aromatic Compound (PAC) Mixtures Containing Active and Inactive Constituents

Kristin M. Eccles[1], Kimberly Gaston[2], Emily M. Green[2], Suramya Waidyanatha[3], Billie Stiffler[4], Shawn F. Harris[5], Cynthia V. Rider[3], Elizabeth Medlock Kakaley[6*]

[1]Environmental Health Science and Research Bureau, Healthy Environments and Consumer Safety Branch, Health Canada, Ottawa, Ontario, K1A 0K9, Canada

[2]Oak Ridge Institute for Science Education, Oak Ridge, Tennessee, 37830, USA

[3]National Institute of Environmental Health Sciences, Division of Translational Toxicology, Durham, North Carolina, 27709, USA

[4]Battelle, Columbus, Ohio 43201, USA

[5]DLH, LLC, Bethesda, Maryland, 20814, USA

[6]U.S Environmental Protection Agency, Center for Public Health and Environmental Assessment, Public Health and Integrated Toxicology Division, Durham, North Carolina, 27709, USA

Table S1. Individual polycyclic aromatic compounds (PACs) used in the current study .

Chemical	CAS	Lot	Purity (%)	Supplier	Top Concentration Tested (mM)
Acenaphthenequinone	82-86-0	MKBW6015V	99.2	Sigma Aldrich	52.1
Benz[*j*]aceanthrylene	202-33-5	AVSC-00148-120817	98.75	AV Square Chem, Inc.	64.6
Benzo[*a*]pyrene	50-32-8	CR66-25-1	>99	MRIGlobal	74.1
Benzo[*b*]fluoranthene	205-99-2	ER03101401	>99.9	Cerilliant Corporation	109
Benzo[*c*]fluorene	205-12-9	JC-OF10-191	>99 by HPLC and 98.3 by GC-MS	Adesis, Inc.	0.19
Benzo[*k*]fluoranthene	207-08-9	501701	>99	Gojira Fine Chemicals, LLC	14.7
Chrysene	218-01-9	1107601	99.86	Gojira Fine Chemicals, LLC	3.29
Dibenz[*a,h*]anthracene	53-70-3	ER032211-01	99.2	Cerilliant Corporation	15.5
Dibenzo[*a,l*]pyrene	191-30-0	AVSC-00140-090916	98.5	AV Square Chem., Inc.	27.1
Dibenzothiophene	132-65-0	BCBN8824V	99.5	Sigma Aldrich	107
Indeno[1,2,3-*cd*]pyrene	193-39-5	SS6-90	99.4	Adesis, Inc.	6.77
Phenanthrene	1985-01-08	7MP2K	98.2	TCI America	108
Pyrene	129-00-0	20151201	98.21	Ivy-Fine Chemicals	108

Table S2. The top concentration of each chemical was used for the equimolar (EM) and Experimental mixtures 1-3 (EXP), for which all serial dilutions were created. All units are in millimolar (mM). The inactive chemicals are bolded.

	ALL			ACTIVES ONLY			
**Chemical**	**EM**	**EXP1**	**EXP2**	**EM**	**EXP1**	**EXP2**	**EXP3**
**Acenaphthenequinone**	**3.85**	**0.13**	**1**	**NA**	**NA**	**NA**	**NA**
Benz[*j*]aceanthrylene	3.84a	0.03a	0.18a	3.84	0.03	0.18	5.29
Benzo[*a*]pyrene	3.84	0.22	0.28	3.84	0.22	0.28	35.9
Benzo[*b*]fluoranthene	3.84	0.21	0.34	3.84	0.21	0.34	6.41
**Benzo[*c*]fluorene**	**0.19b**	**0.19c**	**0.19d**	**NA**	**NA**	**NA**	**NA**
Benzo[*k*]fluoranthene	3.84	0.05	0.05	3.84	0.05	0.05	0.145
**Chrysene**	**3.29e**	**0.005**	**2.12**	**NA**	**NA**	**NA**	**NA**
Dibenz[*a,h*]anthracene	3.85	0.003	0.02	3.85	0.003	0.02	0.105
**Dibenzo[*a,l*]pyrene**	**3.85**	**0.08**	**0.02**	**NA**	**NA**	**NA**	**NA**
**Dibenzothiophene**	**3.85**	**4.06**	**19**	**NA**	**NA**	**NA**	**NA**
Indeno[1,2,3-*cd*]pyrene	3.85	0.22	0.37	3.85	0.22	0.37	2.20
**Phenanthrene**	**3.85**	**13.6**	**14.1**	**NA**	**NA**	**NA**	**NA**
**Pyrene**	**3.85**	**27.6**	**11.5**	**NA**	**NA**	**NA**	**NA**

a) analysis of a mixture solution indicates benz(j)accanthrylene falls out of solution
b) calculated concentration for benzo(c)fluorene EM mixture is 0.832 mg/mL, 3.85 mM. The concentration listed is the maximum solubility of benzo(c)fluorene in DMSO.
c) calculated concentration for benzo(c)fluorene EXP1 mixture is 0.818 mg/mL, 3.78 mM. The concentration listed is the maximum solubility of benzo(c)fluorene in DMSO.
d) calculated concentration for benzo(c)fluorene EXP2 mixture is 0.249 mg/mL, 1.15 mM. The concentration listed is the maximum solubility of benzo(c)fluorene in DMSO
e) calculated concentration for chrysene EM mixture is 0.878 mg/mL, 3.85 mM. The concentration listed is the maximum solubility of benzo(c)fluorene in DMSO.

Table S3. The effective concentration at 10% of maximal response (EC_{10}) used to design the mixing ratio of EXP3.

Chemical	EC_{10}	Std. Error
Benz[*j*]aceanthrylene	0.040	0.029
Benzo[*a*]pyrene	0.272	0.125
Benzo[*b*]fluoranthene	0.049	0.025
Benzo[*k*]fluoranthene	0.001	0.001
Dibenz[*a,h*]anthracene	0.001	0.001
Indeno[*1,2,3-cd*]pyrene	0.017	0.008

Table S4. Parameter constraints for different concentration-response models used in the analysis to meet the different model assumptions. The table specifies whether each parameter, Bottom, Top, Slope, and EC50, is fixed or free for Concentration Addition (CA), Independent Action (IA), and Generalized Concentration Addition (GCA) models. The number in the bracket indicates whether the parameter is fixed.

**Model**	**Bottom**	**Top (%)**	**Slope**	**EC50 (μM)**
Concentration Addition (CA)	Fixed (0)	Fixed (73.02)	Free	Free
Independent Addition (IA)	Fixed (0)	Fixed (73.02)	Free	Free
Generalized Concentration Addition (GCA)	Fixed (0)	Free	Fixed (1)	Free

Table S5. Concentration–response curve parameters for individual PACs used in mixture modeling. Slope refers to the Hill slope of the fitted curve; Top indicates the maximum response as a percentage of the MeBio reference control (%); EC_{50} is the effective concentration (µM) producing 50% of the maximum response. SE = standard error; NA indicates the parameter was fixed during model fitting.

Chemical	Slope	Slope SE	Top	Top SE	EC50	EC50 SE
Benz[*j*]aceanthrylene	0.61	0.09	81.37	NA	2.20	0.53
Benzo[*a*]pyrene	1.10	0.20	81.37	NA	1.83	0.32
Benzo[*b*]fluoranthene	1.12	0.19	81.37	NA	0.52	0.09
Benzo[*k*]fluoranthene	0.86	0.13	73.02	NA	0.05	0.01
Dibenz[*a,h*]anthreacene	0.37	0.06	73.02	NA	0.19	0.07
Indeno[1,2,3-*cd*]pyrene	1.34	0.26	73.02	NA	0.14	0.02
Benz[*j*]aceanthrylene	1	NA	58.08	2.58	0.9	0.19
Benzo[*a*]pyrene	1	NA	80.75	8.21	2.34	0.66
Benzo[*b*]fluoranthene	1	NA	73.51	3.92	0.53	0.11
Benzo[*k*]fluoranthene	1	NA	69.88	2.35	0.04	0.01
Dibenz[*a,h*]anthreacene	1	NA	42.17	2.71	0.01	0
Indeno[1,2,3-*cd*]pyrene	1	NA	79.13	3.93	0.17	0.032

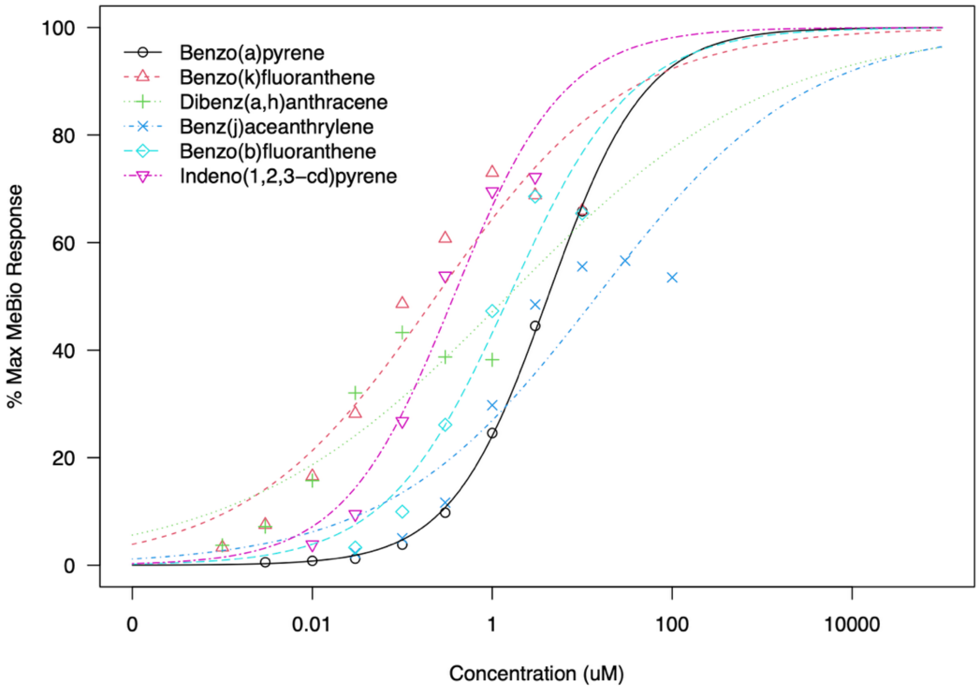

Figure S1. Preliminary concentration–response curves for the individual PACs used to design the EXP3 mixture. Each curve depicts the modeled relationship between chemical concentration and maximal response, with EC10 values corresponding to those reported in Table S1. These curves illustrate the differences in potency when the efficacy is fixed at 100% across components, which informed the proportional mixing scheme applied in EXP3.

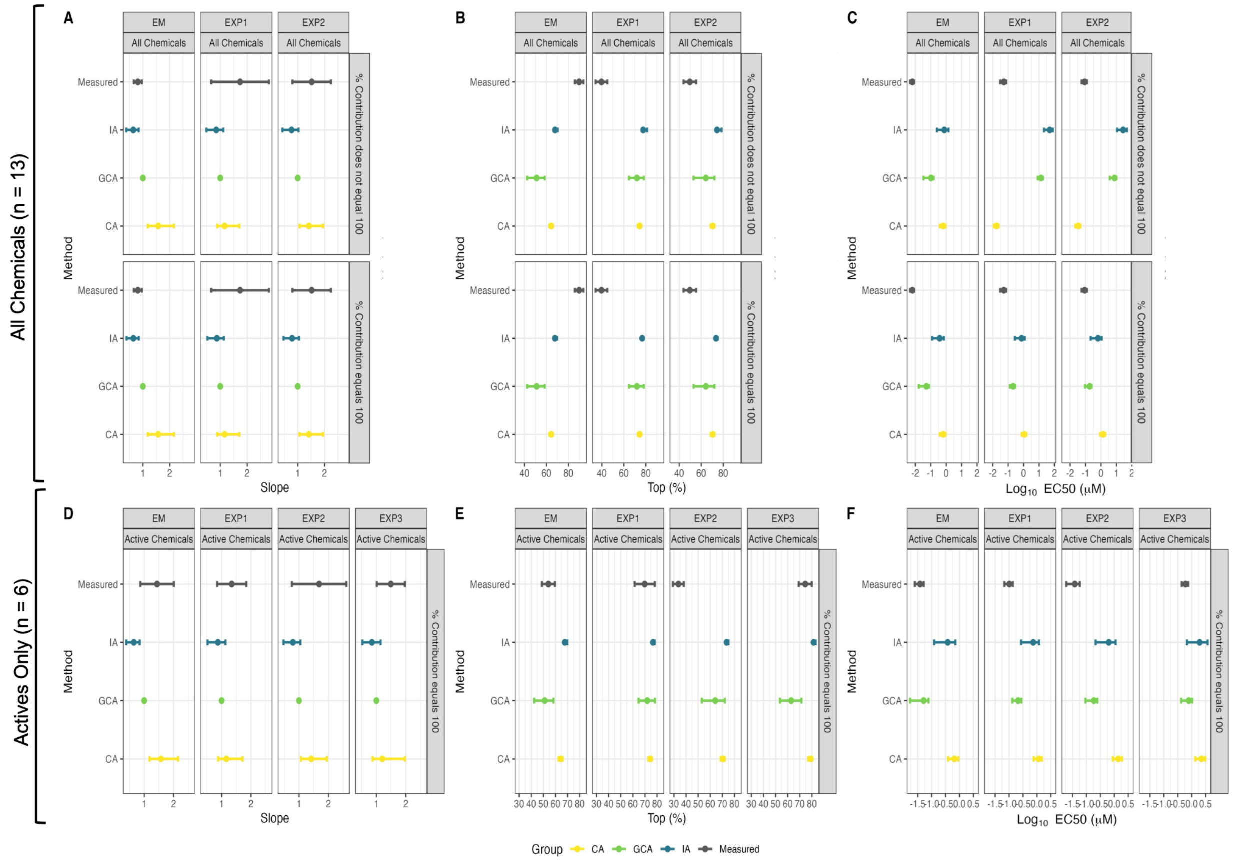

Figure S2. Equimolar mixture (EM), Experimental Mixtures 1 (EXP1), 2 (EXP2), and 3 (EXP3). Panels (A, D) display the estimated slope, the top of the curve (B, E), and EC_{50} values for each mixture corresponding to plots (C, F), which compare measured responses with CA, IA, and GCA predictions. Panels (A-C) correspond to mixtures including all chemicals, while panels (D-F) show results for mixtures containing only active chemicals. Points represent the median, and error bars indicate 95% confidence intervals.
